# An Appy That Needs Epi: An Atypical Presentation of Anaphylaxis

**DOI:** 10.21980/J80H14

**Published:** 2024-01-31

**Authors:** Ryan O’Neill, Cyrus Adeli, Christopher E San Miguel

**Affiliations:** * The Ohio State University, Department of Emergency Medicine, Columbus, OH; ^ The Ohio State University College of Medicine, Clinical Skill Education and Assessment Center, Columbus, OH

## Abstract

**Audience:**

This simulation is intended for 4^th^ year medical students.

**Background:**

Shock is the result of inadequate circulation and failure to perfuse tissues, leading to cellular and organ dysfunction.^1^ Anaphylactic shock specifically is a type of distributive shock secondary to an IgE (immunoglobulin E) dependent reaction, which can result in respiratory compromise and cardiovascular collapse. The National Institute of Allergy and Infectious Diseases/Food Allergy and Anaphylaxis Network (NIAID/FAAN) laid out three diagnostic criteria for the diagnosis of anaphylaxis. Fulfillment of any one of the three following criteria likely indicates anaphylaxis: 1) acute onset of illness with skin findings and either respiratory compromise or reduced blood pressure, 2) involvement of two or more organ systems after exposure to a likely allergen, 3) reduced blood pressure after exposure to a known allergen.^2^ While not a required component of the pathology, hives and cutaneous findings often prompt clinicians to consider anaphylaxis in their differential diagnosis. However, skin findings are absent in 10–20% of cases of anaphylaxis.^3^ It is therefore important for physicians to quickly recognize anaphylactic shock and begin appropriate management in a timely manner even in the absence of skin findings. A previous study of fatal anaphylactic reactions showed a median time to respiratory or cardiac arrest as 30 minutes for foods, 15 minutes for envenomations, and five minutes for iatrogenic reactions.^4^ Drugs are the most common reported cause of fatal anaphylaxis in the United States,^5^ and penicillin allergy is the most common drug allergy reported by patients.^6^ This simulation will help learners recognize an atypical presentation of anaphylactic shock, encourage them to consider anaphylaxis in their differential diagnosis for decompensated patients, and reinforce the correct management of anaphylaxis.

**Educational Objectives:**

At the conclusion of the simulation, learners will be able to: 1) demonstrate ability to efficiently review patient records to optimize patient care and identify relevant details to current presentation, 2) rapidly assess a patient when there is a change in clinical status, 3) recognize the need to start resuscitative fluids for undifferentiated hypotension, 4) identify anaphylaxis, 5) demonstrate the medical management of anaphylaxis, 6) utilize the I-PASS framework to communicate with the inpatient team during the transition of care.

**Educational Methods:**

This summative simulation was designed to assess competence in two of the core Entrustable Professional Activities (EPAs), as defined by the Association of American Medical Colleges (AAMC). These include EPA 8 (Give or Receive a Patient Handover to Transition Care Responsibility) and EPA 10 (Recognize a Patient Requiring Urgent or Emergent Care and Initiate Evaluation and Management). It was performed with 4^th^ year medical students at the conclusion of their month-long emergency medicine (EM) clerkship. This scenario joined seven other scenarios in our pool of potential cases. These sessions are conducted using a high-fidelity manikin as the patient and a confederate/actor in the nursing role. After each scenario concludes, there is a post-simulation debriefing session on the presentation, differential diagnosis, physical exam findings, and management of the target pathology. A Gather-Analyze-Summarize technique was used for the debriefing session.^7^

**Research Methods:**

Facilitators provided informal feedback to the scenario developers after the case was introduced into the assessment rotation. Learners completed a standard evaluation issued by the College of Medicine for the entire session, rather than for individual scenarios. These evaluations were reviewed in aggregate for the first year of implementation. Over this time frame, approximately half the students were run through this scenario.

**Results:**

Overall, our facilitators felt the case fit well into our pool of simulation cases. They felt they were adequately able to assess the students’ ability to respond to a decompensating patient and thought the difficulty level was appropriate for 4^th^ year medical students. The simulation assessment exercise as a whole was highly rated by the students. Of the 198 students who completed an evaluation, 93% rated the overall quality of the session as Very Good or Excellent.

**Discussion:**

Our department has run formative simulations during the 4^th^ year EM clerkship for over ten years. Our primary objective is to assess 4^th^ year students’ competence in EPA 10 (Recognize a Patient Requiring Urgent or Emergent Care and Initiate Evaluation and Management). This case was developed to replace another scenario of anaphylaxis which was felt to be too straightforward and easier than other scenarios in our repertoire. By making the scenario more difficult and the presentation of anaphylaxis a bit atypical, we were able to reinforce the need to include anaphylaxis in the differential diagnosis for any patient who rapidly decompensates. We are also able to review the diagnostic criteria for anaphylaxis and the appropriate treatment, including stopping the exposure to the antigen. This simulation proved to be highly engaging for 4^th^ year medical students, and students seemed to perform at a similar level as previous summative simulations. Overall, we felt this simulation successfully achieved the objectives of the simulation session as a whole, and it was integrated into our 4^th^ year EM clerkship simulation curriculum.

**Topics:**

Medical simulation, emergency medicine, anaphylaxis, anaphylactic shock, allergic reaction, penicillin allergy.

## USER GUIDE

[Table t1-jetem-9-1-s1]

**Table t1-jetem-9-1-s1:** 

List of Resources:	
Abstract	1
User Guide	3
Instructor Materials	5
Operator Materials	22
Debriefing and Evaluation Pearls	29
Simulation Assessment	33
Simulation EPA Assessment	38

**Learner Audience:**

Medical Students

**Time Required for Implementation:**

**Instructor Preparation:** 10 minutes

**Time for case:** 15 minutes

**Time for debriefing:** 20 minutes

**Recommended Number of Learners per Instructor:**

3–4

**Topics:**

Medical simulation, emergency medicine, anaphylaxis, anaphylactic shock, allergic reaction, penicillin allergy.

**Objectives:**

At the conclusion of this simulation, learners will be able to:

1. Demonstrate ability to efficiently review patient records to optimize patient care and identify relevant details to current presentation
2. Rapidly assess a patient when there is a change in clinical status
3. Recognize the need to start resuscitative fluids for undifferentiated hypotension
4. Identify anaphylaxis
5. Demonstrate the medical management of anaphylaxis, including:
  - Stop the offending agent (piperacillin/tazobactam)
  - Administer IM epinephrine
  - Provide supportive care
6. Utilize the I-PASS framework to communicate with the inpatient team during the transition of care

### Linked objectives and methods

Anaphylaxis is a life-threatening and time-sensitive form of shock requiring rapid diagnosis and appropriate management. Learners will be given prior documentation on the patient and have the opportunity to identify relevant details to the current presentation (Objective 1). The patient’s clinical status will change from his initial presentation, enabling learners to initiate assessment of a patient in response to this change (Objective 2). As the patient begins to develop hemodynamic instability, the learners will need to recognize the need to start resuscitative fluids while determining the etiology of the patient’s hypotension. (Objective 3). The learners will then have to identify the presentation of anaphylaxis (Objective 4) and initiate the appropriate treatment (Objective 5). Finally, learners will need to update the admitting team utilizing the IPASS framework (Objective 6).

This scenario has also been designed to assess competence in two of the core Entrustable Professional Activities (EPAs), as defined by the Association of American Medical Colleges (AAMC). These include EPA 8 (Give or Receive a Patient Handover to Transition Care Responsibility) and EPA 10 (Recognize a Patient Requiring Urgent or Emergent Care and Initiate Evaluation and Management). These objectives were tracked by facilitators utilizing an institution-specific EPA evaluation form which is provided as Appendix A. Facilitators used this form to observe critical actions, mark performance, and take notes during the simulation for further discussion during debriefing.

This scenario joined seven other scenarios in our pool of potential cases. At the end of each four-week clerkship, the learners are split into groups of three or four, and they complete three or four simulation scenarios as a team with each learner serving as the team leader once. These sessions are conducted using a high-fidelity manikin as the patient and a confederate/actor in the nursing role. After each scenario concludes, there is a post-simulation debriefing session on the presentation, differential diagnosis, physical exam findings, and management of the target pathology, with this case being anaphylaxis. A Gather-Analyze-Summarize technique was used for the debriefing session.^7^

### Recommended pre-reading for instructor

The instructors should familiarize themselves with the National Institute of Allergy and Infectious Diseases/Food Allergy and Anaphylaxis Network (NIAID/FAAN) diagnostic criteria for anaphylaxis.^2^ They should also review the consensus treatment for anaphylaxis. One good resource for this would be the chapter “Allergy, Hypersensitivity, and Anaphylaxis” from the 9^th^ edition of *Rosen’s Emergency Medicine: Concepts and Clinical Practice*.^8^ Suggested readings include materials listed below in the “References/suggestions for further reading” section.

### Results and tips for successful implementation

This scenario was developed specifically to replace another case of anaphylaxis. In the previous scenario, a patient with a known bee allergy presented to the emergency department with a rash after a bee sting that then progressed to anaphylaxis. The general consensus among facilitators was that the old case did not appropriately challenge learners because the diagnosis was much clearer than in our other simulation scenarios. In order to avoid this same problem, we intentionally did not use moulage to simulate urticaria and only allowed the patient to report mild tongue swelling if specifically asked about this symptom. Since 10–20% of anaphylaxis cases do not involve cutaneous findings, we regarded this atypical presentation as an important learning point rather than an unfair withholding of information from the learners.^3^

Prior to using this scenario as a summative assessment, we ran two rehearsal sessions. The first involved an emergency medicine intern as the sole learner. Initially, the case was written for a patient with no known drug allergies. This seemed to make the scenario significantly more difficult. Based on the first trial run and feedback from experienced facilitators, we decided to include an amoxicillin allergy in the patient’s medical record and include it in the patient’s script if specifically asked about allergies by the learners. The second rehearsal included a group of three 4^th^ year medical students who volunteered to complete an additional simulation case after the completion of their summative simulation session. This group felt the case was similar to the other three scenarios they were given that day and had equally valuable learning points. No substantial changes were made to the case after this second trial run.

Since implementation over a year ago, our facilitators feel the case fits well into our pool of simulation cases. They report being able to adequately assess the students’ ability to respond to a decompensating patient and think the difficulty level is appropriate for 4^th^ year medical students.

As previously described, all 4^th^ year medical students complete a simulation session consisting of three to four scenarios at the conclusion of the EM clerkship. Students are asked to assess the simulation session as a whole, using a standard evaluation form from the College of Medicine. In its first year of use, approximately half of the 4^th^ year students completed the anaphylaxis scenario as part of their simulation session. The simulation session as a whole is highly rated by the students. Of the 198 students who completed an evaluation, 93% rated the overall quality of the session as “very good” or “excellent.” None of the collected comments explicitly mentioned the anaphylaxis case.

## Supplementary Information



## Figures and Tables

**Table t2-jetem-9-1-s1:** 

#1	Handoff report
#2	ED physician documentation
#3	Initial lab results
#4	Radiology results
#5	Medication administration report (MAR)
#6	Repeat CBC
#7	Repeat chemistry
#8	ABG (arterial blood gas)
#9	EKG showing sinus tachycardia
#10	EKG showing ventricular tachycardia
#11	Chest X-ray

**Table t3-jetem-9-1-s1:** 

**Vitals:**	Heart Rate (HR) 115	Blood Pressure (BP) 90/64
	Respiratory Rate (RR) 20	Temperature (T) 99.5°F
	Oxygen Saturation (O_2_Sat) 98% on room air	

**Table t4-jetem-9-1-s1:** 

**Patient Information**		
	**Date of Birth: 6/28/20xx**	**Age: 19**
	**Weight: 180 lbs**.	**MRN: 529374839**
**History of Present Illness**		
**Cody Henderson is a 19 y.o. male who presents with abdominal pain. It started 2 to 3 days ago. Initially, it was generalized, but now has migrated to the RLQ. Has experienced nausea, but no vomiting. No fevers. No diarrhea or constipation, no urinary symptoms. No history of abdominal surgeries**.		
**Patient History**		
**Past Medical: Eczema, ADHD**		**Past Surgical: Right ACL repair (age 17)**
**Medications: Adderall (10mg once daily)**		**Allergies: Amoxicillin**
**Review Of Systems**		
**CNS: non-contributory**		**Renal: non-contributory**
**Cardiovascular: non-contributory**		**Abdominal: abdominal pain started 2 days ago with anorexia**
**Pulmonary: non-contributory**		**Psychiatric: non-contributory**
**Physical Exam**		
**Vitals: 108/78, HR 96, RR 15, O2 98% RA, T 99.5 °C**		
**HEENT: normal**		**Lungs: Clear auscultation bilaterally**
**Neuro: AOx4. Grossly normal**.		**Abdomen: Mild tenderness to RLQ. Positive rebound, no guarding**.
**GU: normal**		**Extremities/MSK: normal**
**Cardiovascular: Regular rate & rhythm. No murmurs, rubs, or gallops**.		**Skin: warm & dry**
**Assessment & Plan**		
**Differential Diagnosis: Appendicitis, viral gastroenteritis. Less likely: renal stone, pyelonephritis**		
**Medical Decision Making:** **Patient presents with migrating pain to RLQ of his abdomen. He has a mild tenderness and rebound on exam. I will obtain basic lab work and CT scan to evaluate for acute appendicitis**. **Clinical Course:** **The patient’s CT scan shows acute uncomplicated appendicitis. He also has a mild leukocytosis. I will consult surgery and start the patient on piperacillin/tazobactam. I anticipate he will be admitted to the hospital for appendectomy**.		

**Table t5-jetem-9-1-s1:** 

Complete Blood Count (CBC)		
Test	Value	Reference Range
**White Blood Cells (WBC) !**	**14.0 × 10**^**3**^**/**μ**L**	**4.5–11.0 × 10**^**3**^**/**μ**L**
**Red Blood Cells (RBC)**	**5.2 × 10**^**6**^**/**μ**L**	**4.5–5.5 × 10**^**6**^**/**μ**L**
**Hemoglobin (Hgb)**	**16 g/dL**	**14–17.4 g/dL**
**Hematocrit (Hct)**	**48%**	**42–52%**
**Platelets**	**340 × 10**^**3**^**/**μ**L**	**150–450 × 10**^**3**^**/**μ**L**

**Table t6-jetem-9-1-s1:** 

Chemistry (Chem-7)		
Test	Value	Reference Range
**Sodium (Na) !**	**131 mmol/L**	**135–145 mmol/L**
**Potassium (K)**	**4.0 mmol/L**	**3.5–5.0 mmol/L**
**Chloride (Cl)**	**96 mmol/L**	**95–105 mmol/L**
**Bicarbonate (CO**_**2**_ **) !**	**20 mmol/L**	**23–29 mmol/L**
**BUN**	**15 mg/dL**	**5–20 mg/dL**
**Creatinine**	**0.8 mg/dL**	**0.6–1.2 mg/dL**
**Glucose**	**95 mg/dL**	**70–100 mg/dL**

**Table t7-jetem-9-1-s1:** 

Medication Name	Dose & Route of Administration	Order Time	Administration Time
**Morphine**	**6 mg IV**	**13:07**	**13:20**
**Ondansetron**	**4 mg IV**	**13:07**	**13:20**
**Normal Saline (0.9% NaCl)**	**1 L IV**	**13:07**	**13:20 – 13:56**
**Iohexol (Omnipaque)**	**38.5 g IV**	**13:15**	**13:35**
**Piperacillin/Tazobactam**	**4.5 g IV**	**14:15**	**15:03 -**

**Table t8-jetem-9-1-s1:** 

Complete Blood Count (CBC)		
Test	Value	Reference Range
**White Blood Cells (WBC) !**	**15.6 × 10**^**3**^**/**μ**L**	**4.5–11.0 × 10**^**3**^**/**μ**L**
**Red Blood Cells (RBC)**	**5.0 × 10**^**6**^**/**μ**L**	**4.5–5.5 × 10**^**6**^**/**μ**L**
**Hemoglobin (Hgb)**	**15.4 g/dL**	**14–17.4 g/dL**
**Hematocrit (Hct)**	**46%**	**42–52%**
**Platelets**	**360 × 10**^**3**^**/**μ**L**	**150–450 × 10**^**3**^**/**μ**L**

**Table t9-jetem-9-1-s1:** 

Chemistry (Chem-7)		
Test	Value	Reference Range
**Sodium (Na) !**	**133 mmol/L**	**135–145 mmol/L**
**Potassium (K)**	**4.2 mmol/L**	**3.5–5.0 mmol/L**
**Chloride (Cl)**	**96 mmol/L**	**95–105 mmol/L**
**Bicarbonate (CO**_**2**_ **) !**	**19 mmol/L**	**23–29 mmol/L**
**BUN**	**13 mg/dL**	**5–20 mg/dL**
**Creatinine**	**0.76 mg/dL**	**0.6–1.2 mg/dL**
**Glucose**	**98 mg/dL**	**70–100 mg/dL**

**Table t10-jetem-9-1-s1:** 

Arterial Blood Gas (ABG)		
Test	Value	Reference Range
**pH !**	**7.3**	**7.35–7.45**
**PaCO**_**2**_	**38 mm Hg**	**35–45 mm Hg**
**PaO**_**2**_	**91 mm Hg**	**80–100 mm Hg**
**HCO**_**3**_ **!**	**19 mm mEq/L**	**22–28 mEq/L**
**O**_**2**_ **Saturation**	**96%**	**92–100%**
**Lactate !**	**2.8 mmol/L**	**< 1.7 mmol/L**

**Figure f1-jetem-9-1-s1:**
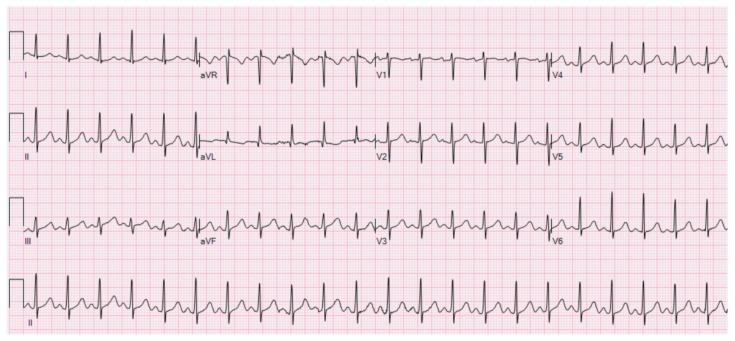


**Figure f2-jetem-9-1-s1:**
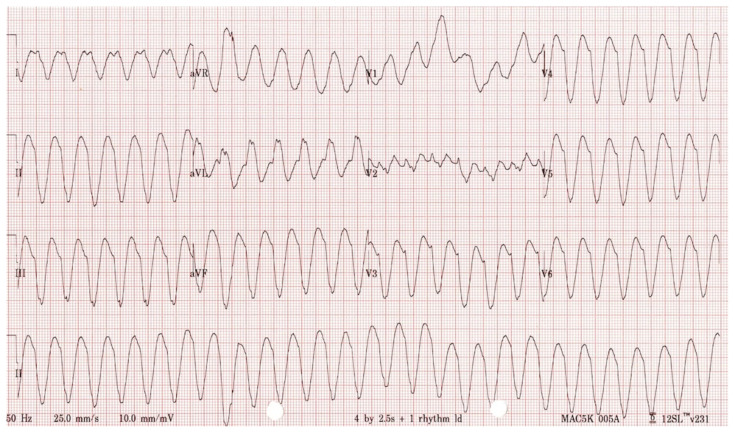


**Figure f3-jetem-9-1-s1:**
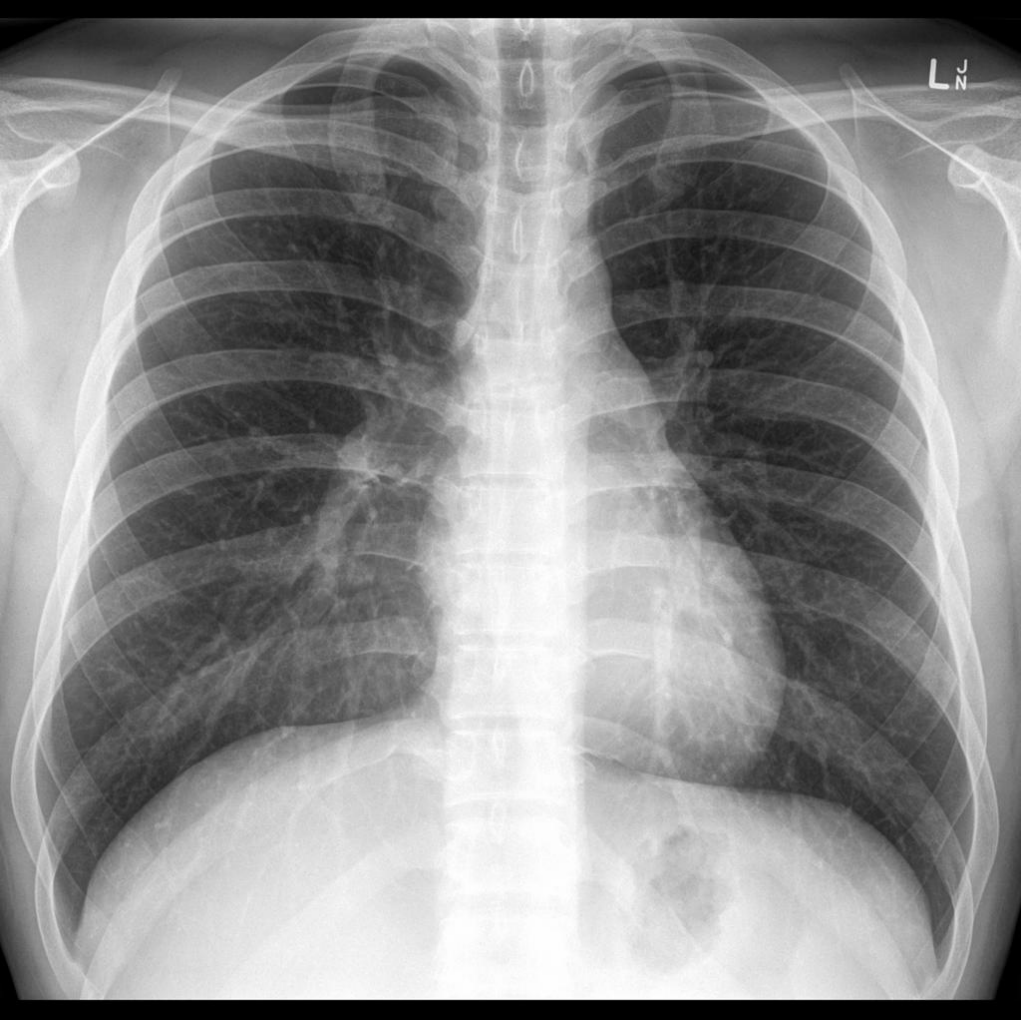


**Table t11-jetem-9-1-s1:** SIMULATION EVENTS TABLE:

Minute (state)	Participant action/trigger	Patient status (simulator response) & operator prompts	Monitor display (vital signs)
State 1 (Baseline)	If IM epinephrine not given in first 5 minutes, go to State 2 (Worsening anaphylaxis) If IM epinephrine given, but piperacillin/tazoba ctam not stopped in first 7 minutes, go to State 2 (Worsening anaphylaxis) If IV code epinephrine given, go to State 4 (V- tach) If IM epinephrine given and piperacillin/tazoba ctam stopped, go to State 5 (Recovery) **Learner Objectives and Interventions**: - Complete Physical Exam - Start resuscitating fluids (ie, NS or LR) - Stop piperacillin/tazobactam - Administer IM epinephrine **Further details**: Learners are expected to either know or look up the **dosages of epinephrine specifically** (and not any other medications). If learners give a dose of IM or subcutaneous epi that is **too little** (ie, **< 0.3 mg**), then proceed like they haven’t given epi. If learners give a dose of IM or subcutaneous epi that is **too large** (ie, **> 0.5 mg**), then proceed as if the patient has received IV code epi, and go to State 4: V-tach. If learners start an **epinephrine drip** or **norepinephrine** (Levophed) drip, then proceed as if they have given **IM epinephrine**. Inaccurate dosages for drips will not affect the state progression, but should be debriefed.	**Sounds/Auscultations**: Mild wheezing bilaterally. “Wheezes” sound effect at 50% volume in every lung speaker. Normal heart and abdominal sounds at normal volume. **Patient Responses**: ○ “I don’t feel good. Feeling lightheaded and nauseated.” ○ “I feel like I need to throw up.” ○ “Started kind of suddenly a few minutes ago.” ○ “Feels like it is getting harder to breathe.” ○ “I’ve never felt like this before.” ○ “My heart is racing.” ○ *Only if specifically asked if there is any swelling or if tongue feels strange:* “Yeah, my tongue does feel a little bigger than normal.” ○ *Only if specifically asked if he has any allergies:* “My mom told me that I got a rash as a kid when I got antibiotics for an ear infection.” Speaking in full sentences, but anxious and scared. Patient’s abdominal pain is similar to original presentation. It had gotten a little better after pain medication, but now it’s back to a 5 out of 10. Onset of symptoms was acute (over minutes). Patient was admitted to the ED two hours ago. No chest pain, rashes, itchiness, headaches, or diarrhea. **Appearance/PE**: Intermittently dry heaving/retching w/no vomit (use sound effects handlers). Eyes wide open, blinking at normal rate. Tongue edema: max.	T 99.5°C HR 115 BP 90/64 RR 20 O2 98% **EKG on monitor**: Sinus Tachycardia
State 2 (Worsening Anaphylaxis)	If either IM epinephrine not given or pip/tazo not stopped in 2 minutes, go to State 3 (PEA) If IV code epinephrine given, go to State 4 (V- tach) If IM epinephrine given and pip/tazo stopped, go to State 5 (Recovery) **Learner Objectives and Interventions**: - Continue resuscitating fluids (ie, NS or LR) - Stop piperacillin/tazobactam - Administer IM epinephrine	**Sounds/Auscultations**: Severe wheezing bilaterally. “Wheezes” sound effect at 80% volume. **Patient Responses**: ○ “It’s getting harder for me to breathe.” ○ “What’s happening to me? Am I going to die?” ○ “My chest feels tight.” ○ “Is this supposed to happen?” **Appearance/PE**: Continue dry heaving. Unwell appearing. Tongue edema: max.	T 99.5° C HR 125 BP 70/50 RR 30 O2 94% End-tidal CO2 (EtCO2) 34 **EKG on monitor**: Sinus Tachycardia
State 3 (PEA)	If 1 minute has passed after epinephrine given (in any form), but piperacillin/tazobactam not stopped, go to State 2 (Worsening anaphylaxis) If epinephrine given (in any form) and piperacillin/tazobactam stopped, go to State 5 (Recovery) If epinephrine (in any form) not given after 3 rounds of ACLS, END CASE **Learner Objectives and Interventions**: - Follows ACLS - Give IV code epinephrine	**Sounds/Auscultations**: Severe wheezing bilaterally. “Wheezes” sound effect at 80% volume. **Patient Responses**: 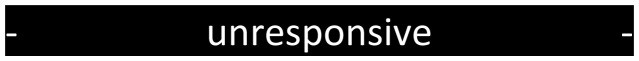 **Appearance/PE**: Eyes closed. Tongue edema: max. **Operator details:** If learners perform adequate compressions, click *“Good Compressions”* handler (sets EtCO2 to 22 and SpO2 to 92%). **Further details**: If learners have still **not recognized** that the patient is having an allergic reaction, have the nurse prompt: “His tongue looks swollen. How do his lungs sound?” If learners **have recognized** that the patent has had an allergic reaction, but they have **still not stopped** piperacillin/tazobactam, have the nurse prompt: “What do you think he is having an allergic reaction to? We don’t use latex anymore.”	T 99.5° C HR 160 BP 0/0 RR 0 O2 0% EtCO2 12 **EKG on monitor**: PEA. Underlying rhythm: sinus tachycardia
State 4 (V-tach)	If patient is cardioverted but piperacillin/tazobactam not stopped, go to State 2 (Worsening anaphylaxis) If 3 minutes in this state elapse, and no cardioversion, go to State 3 (PEA) If cardioversion occurs and piperacillin/tazobactam has been stopped, go to State 5 (Recovery) **Learner Objectives and Interventions**: - Follows ACLS - Synchroninzed cardioversion (minimum 50 J) **Further details**: While learners should deliver a synchronized cardioversion, if learners deliver an unsynchronized cardioversion, proceed as if it were synchronized (for the sake of time). Facilitator should address this mistake during the debrief session.	**Sounds/Auscultations**: Normal lung sounds at normal volume. **Patient Responses**: ○ Patient in an anxious panic for the first 20 seconds: “What’s happening? My heart is racing!” ○ After 20 seconds of being in this state, patient quickly progressively becomes lethargic and can only moan unintelligibly. **Appearance/PE**: Eyes half open. No tongue edema.	T 99.5° C HR 200 BP 60/43 RR 34 O2 92% EtCO2 40 **EKG on monitor**: V-tach *(LLEAP: Ventricular Tachycardia Type 2)*
State 5 (Recovery)	If IM epi given and piperacillin/tazobactam stopped before minute 5, go to Challenge Track If complete handoff to general surgery, go to END CASE **Learner Objectives and Interventions**: - Inform patient of likely allergy - Select new antibiotic for intra-abdominal infection (eg, cipro, flagyl, clinda, ertapenem) - Contact surgical team and give update/handoff	**Sounds/Auscultations**: Normal lung sounds at normal volume **Patient Responses**: “I feel a lot better. What happened?” Patient no longer nauseous, lightheaded, dry heaving, and no longer has difficulty breathing. **Appearance/PE**: Appears well. Eyes wide open. No tongue edema. **Further details**: If learners do not call Gen Surge back on their own, prompt them with the following dialogue: **Nurse:** “The OR called for him. Is he okay to go up now?” “OK, we’ll take him up.” **Facilitator (overhead):** “Hey, this is Gen Surge. I looked up that patient that is coming to the OR, and saw that their antibiotics were stopped earlier. Can you tell me what happened?” “OK. I will update his allergies in the chart. What antibiotic should we put him on instead?”	T 99.5° C HR 108 BP 146/86 RR 14 O2 98% EtCO2 44 **EKG on monitor**: Sinus tachycardia
Challenge Track		If the learner has progressed rapidly through the case, the facilitator can take the patient to State 2: “Worsening Anaphylaxis,” with the goal of having the learner treat **refractory anaphylaxis** with an **epinephrine drip**. This is “bonus” territory, however, and the learner must receive *“Exceeds Expectations*” on their EPA 10 assessment, regardless of performance after this point. Ideal management of refractory anaphylaxis is an epinephrine drip. They should start the IV epinephrine infusion at 0.1 mcg/kg/minute (range: 0.05 to 0.2 mcg/kg/minute) and increase it every two to three minutes by 0.05 mcg/kg/minute until BP and perfusion improve.	

**Table t12-jetem-9-1-s1:** 

**I**	Illness Severity	Stable
**P**	Patient Summary	This patient initially presented with RLQ pain and was found to have appendicitis. He had an anaphylactic reaction to the piperacillin/ tazobactam drip, requiring IM epinephrine. He is now stable.
**A**	Action List	He requires ongoing monitoring as he is receiving his new antibiotic regimen. He ultimately still requires operative management of his appendicitis.
**S**	Situation Awareness and Contingency Planning	If patient has another episode of hypotension/anaphylaxis, he will require re-dosing of IM epinephrine, consideration of an epinephrine drip, and possible search for another trigger.
**S**	Synthesis by Receiver	From the facilitator: *“So, this patient has appendicitis and a new apparent anaphylactic allergy to piperacillin/tazobactam that required IM epinephrine. He is stable now, but requires ongoing monitoring and surgery for his appendicitis.”*

**Table t13-jetem-9-1-s1:** 

**Critical Actions:** Performs a focused history and physical exam (including obtaining vital signs) Gives IV Fluid Bolus (LR or NS acceptable) Reviews the existing medical record Stops piperacillin/tazobactam infusion Gives diphenhydramine Gives 0.3–0.5 mg epinephrine SQ or IM (no credit for IV) Updates the admitting surgical team	0:00

**Table t14-jetem-9-1-s1:** 

	Milestone	Did not achieve level 1	Level 1	Level 2	Level 3
1	**Emergency Stabilization (PC1)**	□ Did not achieve Level 1	□ Recognizes abnormal vital signs	□ Recognizes an unstable patient, requiring intervention Performs primary assessment Discerns data to formulate a diagnostic impression/plan	□ Manages and prioritizes critical actions in a critically ill patient Reassesses after implementing a stabilizing intervention
2	**Performance of focused history and physical (PC2)**	□ Did not achieve Level 1	□ Performs a reliable, comprehensive history and physical exam	□ Performs and communicates a focused history and physical exam based on chief complaint and urgent issues	□ Prioritizes essential components of history and physical exam given dynamic circumstances
3	**Diagnostic studies (PC3)**	□ Did not achieve Level 1	□ Determines the necessity of diagnostic studies	□ Orders appropriate diagnostic studies. Performs appropriate bedside diagnostic studies/procedures	□ Prioritizes essential testing Interprets results of diagnostic studies Reviews risks, benefits, contraindications, and alternatives to a diagnostic study or procedure
4	**Diagnosis (PC4)**	□ Did not achieve Level 1	□ Considers a list of potential diagnoses	□ Considers an appropriate list of potential diagnosis May or may not make correct diagnosis	□ Makes the appropriate diagnosis Considers other potential diagnoses, avoiding premature closure
5	**Pharmacotherapy (PC5)**	□ Did not achieve Level 1	□ Asks patient for drug allergies	□ Selects an medication for therapeutic intervention, consider potential adverse effects	□ Selects the most appropriate medication and understands mechanism of action, effect, and potential side effects Considers and recognizes drug-drug interactions
6	**Observation and reassessment (PC6)**	□ Did not achieve Level 1	□ Reevaluates patient at least one time during case	□ Reevaluates patient after most therapeutic interventions	□ Consistently evaluates the effectiveness of therapies at appropriate intervals
7	**Disposition (PC7)**	□ Did not achieve Level 1	□ Appropriately selects whether to admit or discharge the patient	□ Appropriately selects whether to admit or discharge Involves the expertise of some of the appropriate specialists	□ Educates the patient appropriately about their disposition Assigns patient to an appropriate level of care (ICU/Tele/Floor) Involves expertise of all appropriate specialists
9	**General Approach to Procedures (PC9)**	□ Did not achieve Level 1	□ Identifies pertinent anatomy and physiology for a procedure Uses appropriate Universal Precautions	□ Obtains informed consent Knows indications, contraindications, anatomic landmarks, equipment, anesthetic and procedural technique, and potential complications for common ED procedures	□ Determines a back-up strategy if initial attempts are unsuccessful Correctly interprets results of diagnostic procedure
20	**Professional Values (PROF1)**	□ Did not achieve Level 1	□ Demonstrates caring, honest behavior	□ Exhibits compassion, respect, sensitivity and responsiveness	□ Develops alternative care plans when patients’ personal beliefs and decisions preclude standard care
22	**Patient centered communication (ICS1)**	□ Did not achieve level 1	□ Establishes rapport and demonstrates empathy to patient (and family) Listens effectively	□ Elicits patient’s reason for seeking health care	□ Manages patient expectations in a manner that minimizes potential for stress, conflict, and misunderstanding. Effectively communicates with vulnerable populations, (at risk patients and families)
23	**Team management (ICS2)**	□ Did not achieve level 1	□ Recognizes other members of the patient care team during case (nurse, techs)	□ Communicates pertinent information to other healthcare colleagues	□ Communicates a clear, succinct, and appropriate handoff with specialists and other colleagues Communicates effectively with ancillary staff

## INSTRUCTOR MATERIALS

**Case Title:** Penicillin-Induced Anaphylaxis

**Case Description & Diagnosis (short synopsis):** This scenario presents an atypical case of anaphylaxis. This case involves an established ED patient with a known diagnosis of appendicitis that the learners receive in sign out. The scenario begins after the patient has been diagnosed with appendicitis, started on piperacillin/tazobactam, and is waiting to go to the operating room. While the initial complaint of not feeling well, nausea, and right lower quadrant (RLQ) abdominal pain could be caused by worsening appendicitis, the patient will have wheezing, which will exacerbate throughout the case. This, along with the rapidity of clinical decline, is the key aspect leading to the correct diagnosis. With tongue swelling (which will likely be difficult for learners to appreciate on the manikin), wheezing, dry heaving, and hypotension, there is clear indication for the use of epinephrine. The offending agent, piperacillin/tazobactam, also needs to be stopped. If the learner gives epinephrine without stopping the antibiotic, the patient will continue to decompensate. Another key learning point is the use of IM or subcutaneous epinephrine, rather than IV epinephrine, to treat anaphylaxis. If given IV epinephrine, the patient will go into an unstable ventricular tachycardia, requiring cardioversion. The ideal management would be the following: Obtain vital signs, perform history and physical exam, review the medical record, identify anaphylaxis, stop the piperacillin/tazobactam infusion, and give IM epinephrine.

**Equipment or Props Needed:**

- High fidelity simulator (this study used a SimMan® 3G Manikin)
- Hospital-grade defibrillator
- Cardiac monitor
- Pulse oximetry
- IV pole
- Peripheral IV line
- Crash cart with ambu-bag and intubation kit
- Non-rebreather mask
- Nasal cannula
- Zosyn bag (100 ml) (see Appendix C)
- IV Pump
- Epi auto-injector (at most 2)
- “Demo Dose” Epi boxes
- Nebulizer mask w/oxygen tubing (adult). For delivery of aerosol medications, such as albuterol or DuoNeb. Insert water into the chamber for effect.
- Three 1 L bags of fluid (either NS or LR)
- Other simulated medications with labeling (not limited to: epinephrine syringe epinephrine bag, diphenhydramine, methylprednisolone, levophed, albuterol, duoneb, prednisone tablet)

**Actors needed:**

One actor or confederate in the nursing role and the facilitator to provide the initial patient handoff as the off-going emergency physician and then to accept handoff at the end of the case as the surgery attending.

**Stimulus Inventory:** [Table t2-jetem-9-1-s1]

**Background and brief information:** The patient is one of four patients being given to the learner in sign-out in the emergency department. He is a 19-year-old male who presented with RLQ abdominal pain, and his computerized tomography (CT) showed simple uncomplicated appendicitis. He’s been started on antibiotics and is waiting to be taken to surgery. The facilitator begins the scenario by entering the learner waiting area and saying the following: “Hey, I’m glad you’re here. I need to get out of here. Can I tell you about a couple of patients?” The facilitator will then hand the learner a list of patients and briefly talk about each patient. They will talk fast and not take questions. After the learners have 30 seconds to read the handoff report, prompt the nurse to interrupt their conversation in the hallway and bring the learners into the bay, while asking them to come assess the patient.

**Initial presentation:** The patient is overall well-appearing, but intermittently dry heaving. Eyes are wide open. He is speaking in full sentences, but anxious and scared. He is complaining of nausea, lightheadedness, shortness of breath, and heart racing. The patient’s abdominal pain is similar to his original presentation. It had gotten a little better after pain medication, but now it’s back to a five out of ten. The onset of symptoms was acute (over a few minutes). The patient was admitted to the ED two hours ago. He has no chest pain, rashes, itchiness, headaches, or diarrhea.

**How the scene unfolds:** This case starts with an established ED patient with a known diagnosis of appendicitis. The learners receive a rushed handoff from the off-going emergency physician, who gives them four potential patients. Shortly after receiving this handoff, the nurse asks the learners to come assess the patient because he is not feeling well. If asked any questions about the patient’s history, the nurse will only tell them that she is covering for a colleague on lunch, and all she knows is that he has appendicitis. There are a few ways in which the learners can identify anaphylaxis: clinically, via his rapid decline and wheezing, or ask the patient about his known allergies, or review the medical record which lists his allergy to amoxicillin. If the piperacillin/tazobactam is stopped, and the patient receives IM or subcutaneous epinephrine, he will recover. If at any point the patient is given IV epinephrine, he will go into ventricular tachycardia, requiring cardioversion to recover. If the patient does not receive epinephrine and/or the piperacillin/tazobactam isn’t stopped, his condition will worsen until he ultimately suffers a PEA cardiac arrest. The case ultimately ends with a verbal handoff to general surgery.

**Critical actions:**

1. Performs a focused history and physical exam (including obtaining vital signs)
2. Reviews the existing medical record
3. Gives IV Fluid Bolus (LR or NS acceptable)
4. Gives diphenhydramine
5. Stops piperacillin/tazobactam infusion
6. Gives 0.3–0.5 mg epinephrine SQ or IM (no credit for IV)
7. Updates the admitting surgical team

**Case Title:** Penicillin-Induced Anaphylaxis

**Chief Complaint:** Right lower quadrant (RLQ) abdominal pain. The patient is a 19-year-old male who presented with RLQ abdominal pain. His CT showed simple uncomplicated appendicitis. He’s been started on antibiotics and is waiting to be taken to surgery.[Table t3-jetem-9-1-s1]

**General Appearance:** Alert and interactive, intermittently dry heaving without vomiting

**Primary Survey:**

- **Airway:** Speaking in full sentences
- **Breathing:** Diffuse mild wheezing bilaterally
- **Circulation:** 2+ pulses to all extremities

**History:**

- **History of present illness:** 19 y.o. male who presents with abdominal pain. It started two to three days ago. Initially, it was generalized, but now has migrated to the RLQ. Has experienced nausea, but no vomiting. No fevers. No diarrhea or constipation, no urinary symptoms.
- **Past medical history:** Eczema, attention deficit/hyperactivity disorder (ADHD)
- **Past surgical history:** None
- **Patient’s medications:** Adderall (10mg once daily)
- **Allergies:** Amoxicillin
- **Social history:** None
- **Family history:** None

**Secondary Survey/Physical Examination:**

**General Appearance**: Alert and interactive, intermittently dry heaving without vomiting

- **HEENT**
  ○ Head: within normal limits
  ○ Eyes: within normal limits
  ○ Ears: within normal limits
  ○ Nose: within normal limits
  ○ Throat/oropharynx: tongue edema
- **Neck**: within normal limits
- **Heart**: tachycardic
- **Lungs**: mild diffuse wheezes bilaterally
- **Abdominal/GI**: soft with mild rlq pain and positive rebound, unchanged from arrival
- **Genitourinary**: deferred/within normal limits
- **Rectal**: within normal limits
- **Extremities**: within normal limits
- **Neuro**: within normal limits
- **Skin**: within normal limits.
- **Lymph**: within normal limits
- **Psych**: within normal limits

*Handoff Report*

**Handoff from ED attending**

**Room 13:**

**Mary Morrison is a 65-year-old female who presented with left-sided chest pain. She is stable-appearing and her EKG is non-diagnostic. Given her risk factors, if her troponin is negative, she will need to be placed in observation for a stress test tomorrow**.

**Room 25:**

**Cody Henderson is a 19-year-old male who presented with RLQ abdominal pain. His CT showed simple uncomplicated appendicitis. He’s been started on antibiotics and is waiting to be taken to surgery**.

**Room 18:**

**Jonathon Lemming is a 25-year-old male who presented with right ankle pain after rolling it while playing soccer. He is pending an x-ray of the ankle. If it shows a fracture, he will need an orthopedic surgery consult. If the x-ray is negative, he will need an ace wrap, crutches, and discharge as an ankle sprain**.

**Room 7:**

**Henrietta Jones is a 78-year-old female who presented with cough and fever. She was hypoxic on arrival and is now on heated high flow. She is COVID+ and will go to the ICU when a bed is made available**.

*ED Physician Documentation*

**Documentation from ED Physician** [Table t4-jetem-9-1-s1]

*Initial CBC* [Table t5-jetem-9-1-s1]

*Initial Chemistry* [Table t6-jetem-9-1-s1]

*CT results*

**Radiology Results**

**EXAM: CT ABDOMEN/PELVIS WITH CONTRAST, 13:35 PM**

**CLINICAL INDICATIONS: RLQ Abdominal Pain**

**TECHNIQUE: CT images of the abdomen and pelvis were performed from the lung bases through the ischial tuberosities following the administration of intravenous contrast. CONTRAST: Iohexol (OMNIPAQUE) 350 MG/ML injection 1–171 mL; Route of Administration: Intravenous; Dose: 110 mL**.

**FINDINGS:**

- **Lung Bases: The visualized lung bases and lower mediastinal structures are unremarkable**.
- **Liver: Liver is normal in size. No focal lesions**.
- **Biliary/Gallbladder: The gallbladder is normal in size without evidence of stones or sludge. There is no pericholecystic fluid. The biliary tree is nondilated**.
- **Spleen: Spleen is normal in size and CT density**.
- **Pancreas: Pancreas is normal. There is no evidence of pancreatic mass or peripancreatic fluid**.
- **Adrenals: Adrenal glands are unremarkable**.
- **Kidneys: Kidneys are normal in size. There are no stones or hydronephrosis**.
- **Retroperitoneal/Vasculature: No retroperitoneal adenopathy is identified**.
- **Gastrointestinal/Mesentery: Increased appendiceal caliber with diameter of 1.1 cm with wall thickening up to 5 mm and adjacent fat stranding. Multiple enlarged lymph nodes surrounding the appendix. No adjacent extraluminal collection of gas or pus. The bowel loops are non-dilated without wall thickening or mass**.
- **Bladder: The bladder is normal**.
- **Genital: The prostate and seminal vesicles are unremarkable**.
- **Bony Structures: Visualized bony structures are consistent with the patient’s age**.

**IMPRESSION:**

1. **Acute uncomplicated appendicitis. No CT evidence of perforation. This critical finding has been discussed with the ED Attending with a read back at 1405**.

*Medication Administration Report (MAR)* [Table t7-jetem-9-1-s1]

*Repeat CBC* [Table t8-jetem-9-1-s1]

*Repeat Chemistry* [Table t9-jetem-9-1-s1]

*ABG* [Table t10-jetem-9-1-s1]

*EKG showing sinus tachycardia*

Ewingdo. ECG Sinus Tachycardia 132 bpm. In: Wikimedia Commons.

https://en.wikipedia.org/wiki/File:ECG_Sinus_Tachycardia_132_bpm.jpg Published November 13, 2020. Accessed November 18, 2023. CC BY-SA 4.0.[Fig f1-jetem-9-1-s1]

*EKG showing ventricular tachycardia*

Buttner R, Burns E. Monomorphic ventricular tachycardia. In: Life in the Fast

Lane. https://litfl.com/ventricular-tachycardia-monomorphic-ecg-library. CC BY-NC-SA 4.0.[Fig f2-jetem-9-1-s1]

*Chest X-ray*

Gaillard, Frank. Normal frontal chest x-ray. In:

Radiopaedia. https://radiopaedia.org/cases/normal-frontal-chest-x-ray. CC BY-NC-SA 3.0.[Fig f3-jetem-9-1-s1]

## OPERATOR MATERIALS

[Table t11-jetem-9-1-s1]

**Diagnosis:**

Penicillin-induced anaphylaxis

**Disposition:**

Admission to surgery

## DEBRIEFING AND EVALUATION PEARLS

### An Appy That Needs Epi: An Atypical Presentation of Anaphylaxis

**Pearls:**

1. **Demonstrate ability to efficiently review patient records to optimize patient care and identify relevant details to current presentation**
  - When available, previous records can be very helpful when there is a change in clinical status. They can provide details that the patient is now unable to provide, insight into previous provider’s medical decision making, and old (but still relevant) test results. Don’t over-rely on these records, however, as you want to avoid a “momentum bias” (that is, simply continuing the same thought process as the previous provider).
2. **Rapidly assess a patient when there is a change in clinical status**
  - Any significant change in status should necessitate an in-person bedside evaluation.
  - As always, first assess “ABC’s.”
  - Focus on what has changed since the last assessment.
  - If old information is not available or doesn’t make sense, consider restarting the encounter and assessment entirely.
3. **Recognize the need to start resuscitative fluids for undifferentiated hypotension**
  - This should be the default treatment for hypotension. Fluids can help temporize hypotension while you figure out the underlying cause.
  - Fluids should be balanced crystalloid. Use LR or NS. 500 mL to 1000 mL.
  - Take caution in patients with heart failure, renal failure, liver failure, or evidence of volume overload on exam.
4. **Identify anaphylaxis**
  - Three different possible criteria (only need to meet one)
    - Acute onset of illness (minutes to hours) involving skin, mucosal tissue, or both, and one of the following: respiratory compromise or hypotension.
    - Two or more of the following occurring rapidly after exposure to a likely allergen: involvement of the skin-mucosa, respiratory compromise, reduced BP, or persistent GI symptoms and signs.
    - Reduced BP after exposure to a known allergen.
  - General rule of thumb: classic allergy symptoms affecting two systems or causing hypotension.
5. **Demonstrate the medical management of anaphylaxis, including:**
  - Stop the offending agent (piperacillin/tazobactam).
  - Administer IM epinephrine.
    - Even in many healthcare settings, auto-injector is the preferable method of delivery, for it avoids dosing errors and allows the fastest delivery.
    - IM is preferred over IV because of decreased cardiovascular complications such as severe hypertension and ventricular arrhythmia.
    - In anaphylaxis, there are no absolute complications to epinephrine use, but should be used in caution in certain populations.
      1. Patients with cardiovascular disease
      2. Patients with conditions sensitive to hypertension (eg, recent intracranial surgery, aortic aneurysm)
      3. Patients using stimulants
    - EpiPen autoinjector delivers 0.3 mg IM, EpiPen Jr delivers 0.15 mg IM.
    - If drawing up dose, give 0.01 mg/kg (up to a maximum of 0.5 mg).
    - If there is an inadequate response, dosing can be repeated every 5–10 minutes.
    - If requiring a second dose, you should be prepared to start an epinephrine drip if a third dose is required.
      1. Starting dose is 0.1 mcg/kg/min and should be increased every 2–3 minutes by .05 mcg/kg/min to effect.
      2. If peri-code, can start higher or titrate up faster.
    - As of 2016, epinephrine 1:1000 injection is now only labeled as 1 mg/mL, while epinephrine 1:10,000 injection is only labeled as 0.1 mg/mL.
    - If IM concentration of epinephrine is not available, IV bolus epinephrine can be given, but the dose is reduced to 0.05–0.1 mg.
  - Provide Supportive Care
    - IV Fluid bolus can be very helpful because there can be a large fluid shift to the extravascular space during anaphylaxis.
    - Antihistamines (both H1 and H2 blockers) can reduce itching and hives, but they do not treat any other aspects of anaphylaxis (such as airway obstruction and hypotension).
    - Bronchodilators can be helpful in treating bronchospasm associated with anaphylaxis, but should be used with epinephrine not in place of epinephrine.
    - Glucocorticoids are controversial in treating anaphylaxis. Their onset of action is in the order of hours, so if they do anything, it is to prevent the second half of a biphasic reaction. However, the evidence does not show that they are even particularly good at that.
6. **Utilize the I-PASS framework to communicate with the inpatient team during the transition of care**
  - Illness severity – Describe whether the patient is stable, unstable, or someone that may decompensate.
  - Patient Summary – Give a summary statement and explain the hospital course up until now.
  - Action List – Explain what still needs to be done for the patient.
  - Situation awareness and contingency plans – Highlight any potential changes that may occur in the patient’s clinical status and what could be done if they were to happen.
  - Synthesis by Receiver-Utilizing closed loop communication, the receiver explains their understanding of the situation to the one giving sign-out.
  - When reflecting on the sign-out given at the beginning of this case, one can see how unhelpful it was for understanding what is happening with the patient. When looking at the example below, you can understand why this framework is a much more effective form of communication.

#### Exemplar I-PASS Sign-Out For Cody Henderson

[Table t12-jetem-9-1-s1]

## SIMULATION ASSESSMENT

*Penicillin-Induced Anaphylaxis*

Learner: _________________________________________

***Assessment Timeline***

This timeline is to help observers assess their learners. It allows observer to make notes on when learners performed various tasks, which can help guide debriefing discussion.[Table t13-jetem-9-1-s1]

**Critical Actions:**

□ Performs a focused history and physical exam (including obtaining vital signs)
□ Gives IV Fluid Bolus (LR or NS acceptable)
□ Reviews the existing medical record
□ Stops piperacillin/tazobactam infusion
□ Gives diphenhydramine
□ Gives 0.3–0.5 mg epinephrine SQ or IM (no credit for IV)
□ Updates the admitting surgical team

**Summative and formative comments:**

**Milestones assessment:** [Table t14-jetem-9-1-s1]

**EPA 10 Assessment**

1. Performs a focused history and physical exam (including obtaining vital signs)
  ○ No
  ○ Incorrectly or incompletely
  ○ Yes, with prompting
  ○ Yes
2. Gives IV Fluid Bolus (LR or NS is acceptable)
  ○ No
  ○ Incorrectly or incompletely
  ○ Yes, with prompting
  ○ Yes
3. Reviews the existing medical record
  ○ No
  ○ Incorrectly or incompletely
  ○ Yes, with prompting
  ○ Yes
4. Stops piperacillin/tazobactam infusion.
  ○ No
  ○ Incorrectly or incompletely
  ○ Yes, with prompting
  ○ Yes
5. Gives 0.3–0.5 mg epinephrine SQ or IM (no credit for IV)
  ○ No
  ○ Incorrectly or incompletely
  ○ Yes, with prompting
  ○ Yes
6. Gives diphenhydramine
  ○ No
  ○ Incorrectly or incompletely
  ○ Yes, with prompting
  ○ Yes
7. Updates the admitting surgical team
  ○ No
  ○ Incorrectly or incompletely
  ○ Yes, with prompting
  ○ Yes
8. Did this student perform any dangerous actions?
  ○ No
  ○ Yes
9. Additional Comments
10. Would you feel confident in this student’s ability to manage an acutely decompensating/acutely ill patient with a life-threatening illness?
  ○ No
  ○ Yes
11. Additional Comments Regarding EPA 10

**EPA 8 Assessment**

1. Did the student who gave the handoff provide the following: Illness Severity
  ○ No
  ○ Incompletely
  ○ Yes
2. Did the student who gave the handoff provide the following: Patient Summary
  ○ No
  ○ Incompletely
  ○ Yes
3. Did the student who gave the handoff provide the following: Action List
  ○ No
  ○ Incompletely
  ○ Yes
4. Did the student who gave the handoff provide the following: Situational Awareness/Contingency Planning
  ○ No
  ○ Incompletely
  ○ Yes
5. Did the student who gave the handoff provide the following: Synthesis by Receiver
  ○ No
  ○ Incompletely
  ○ Yes
6. Would you feel confident in this student’s ability to give patient handover to transition care responsibility?
  ○ No
  ○ Yes
7. Additional Comments Regarding EPA 8
