# Supplementary material for: An Appy That Needs Epi: An Atypical Presentation of Anaphylaxis
Source: J Educ Teach Emerg Med. 2024 Jan 31;9(1):S1–S41. doi: 10.21980/J80H14 (PMC10854882; doi:10.21980/J80H14)

## Slide 1
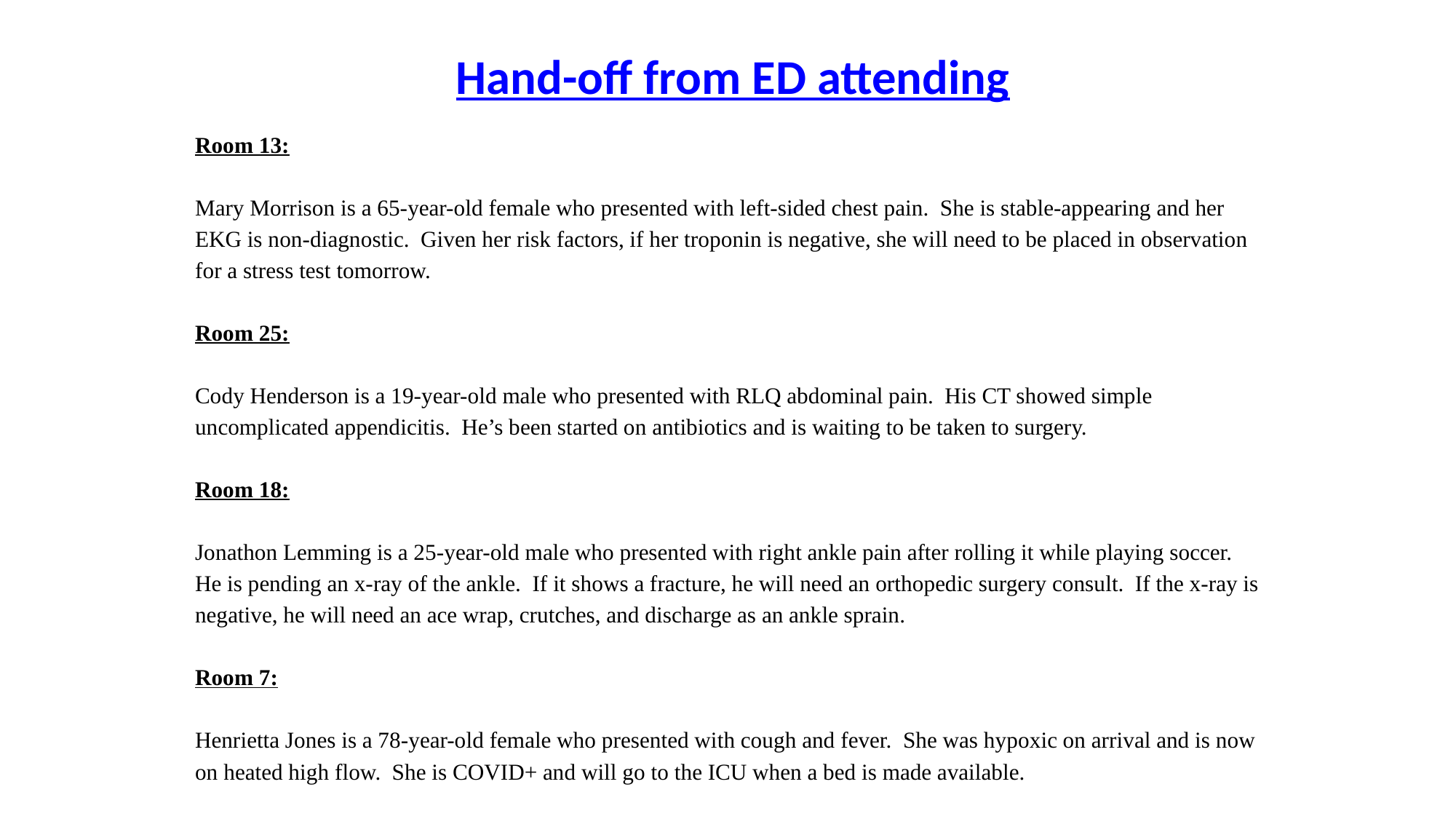

Hand-off from ED attending
Room 13:
Mary Morrison is a 65-year-old female who presented with left-sided chest pain. She is stable-appearing and her EKG is non-diagnostic. Given her risk factors, if her troponin is negative, she will need to be placed in observation for a stress test tomorrow.
Room 25:
Cody Henderson is a 19-year-old male who presented with RLQ abdominal pain. His CT showed simple uncomplicated appendicitis. He’s been started on antibiotics and is waiting to be taken to surgery.
Room 18:
Jonathon Lemming is a 25-year-old male who presented with right ankle pain after rolling it while playing soccer. He is pending an x-ray of the ankle. If it shows a fracture, he will need an orthopedic surgery consult. If the x-ray is negative, he will need an ace wrap, crutches, and discharge as an ankle sprain.
Room 7:
Henrietta Jones is a 78-year-old female who presented with cough and fever. She was hypoxic on arrival and is now on heated high flow. She is COVID+ and will go to the ICU when a bed is made available.

## Slide 2
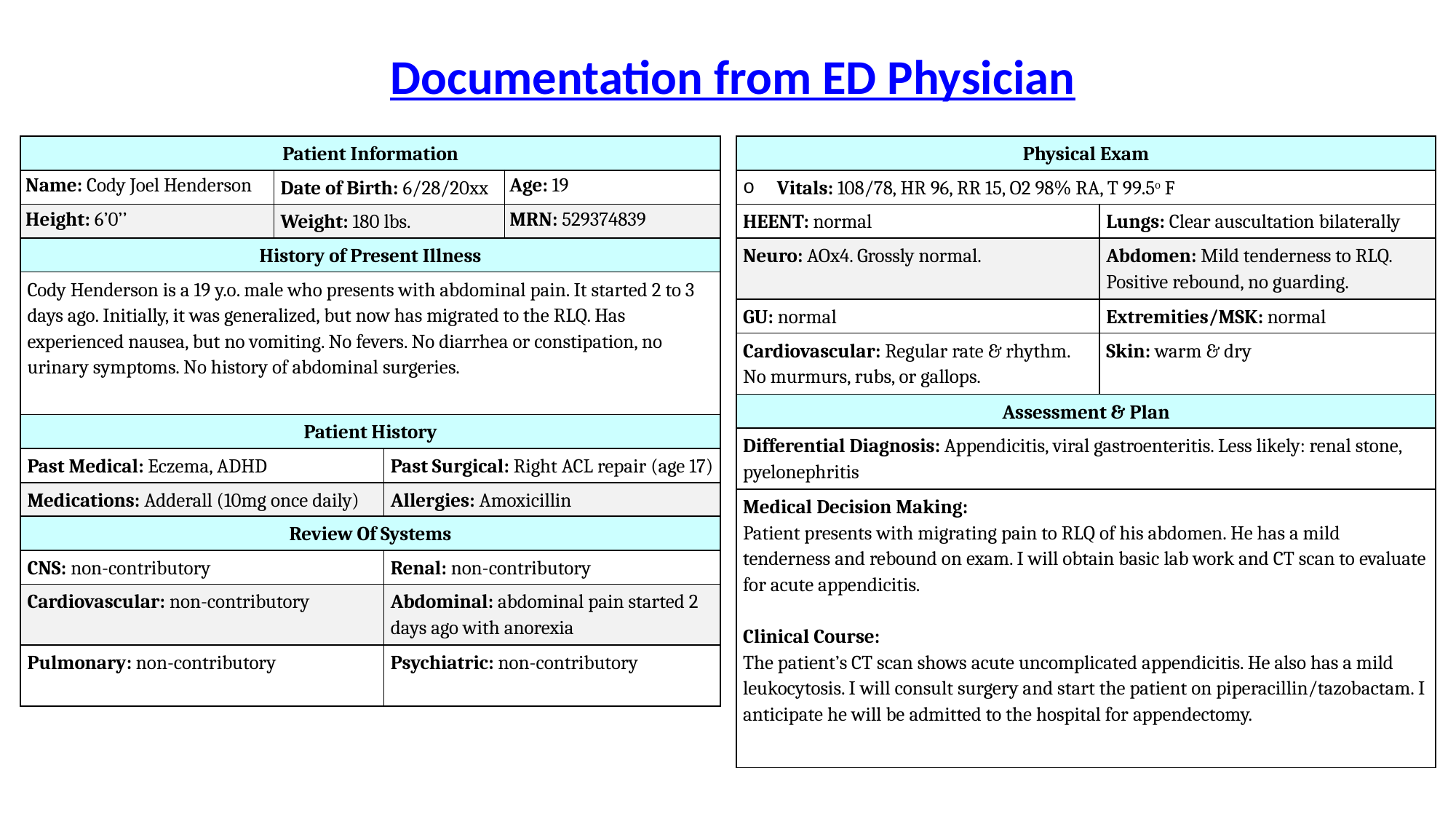

Documentation from ED Physician
| Patient Information | | | |
| --- | --- | --- | --- |
| Name: Cody Joel Henderson | Date of Birth: 6/28/20xx | | Age: 19 |
| Height: 6’0’’ | Weight: 180 lbs. | | MRN: 529374839 |
| History of Present Illness | | | |
| Cody Henderson is a 19 y.o. male who presents with abdominal pain. It started 2 to 3 days ago. Initially, it was generalized, but now has migrated to the RLQ. Has experienced nausea, but no vomiting. No fevers. No diarrhea or constipation, no urinary symptoms. No history of abdominal surgeries. | | | |
| Patient History | | | |
| Past Medical: Eczema, ADHD | | Past Surgical: Right ACL repair (age 17) | |
| Medications: Adderall (10mg once daily) | | Allergies: Amoxicillin | |
| Review Of Systems | | | |
| CNS: non-contributory | | Renal: non-contributory | |
| Cardiovascular: non-contributory | | Abdominal: abdominal pain started 2 days ago with anorexia | |
| Pulmonary: non-contributory | | Psychiatric: non-contributory | |
| Physical Exam | |
| --- | --- |
| Vitals: 108/78, HR 96, RR 15, O2 98% RA, T 99.5o F | |
| HEENT: normal | Lungs: Clear auscultation bilaterally |
| Neuro: AOx4. Grossly normal. | Abdomen: Mild tenderness to RLQ. Positive rebound, no guarding. |
| GU: normal | Extremities/MSK: normal |
| Cardiovascular: Regular rate & rhythm. No murmurs, rubs, or gallops. | Skin: warm & dry |
| Assessment & Plan | |
| Differential Diagnosis: Appendicitis, viral gastroenteritis. Less likely: renal stone, pyelonephritis | |
| Medical Decision Making: Patient presents with migrating pain to RLQ of his abdomen. He has a mild tenderness and rebound on exam. I will obtain basic lab work and CT scan to evaluate for acute appendicitis.   Clinical Course: The patient’s CT scan shows acute uncomplicated appendicitis. He also has a mild leukocytosis. I will consult surgery and start the patient on piperacillin/tazobactam. I anticipate he will be admitted to the hospital for appendectomy. | |

## Slide 3
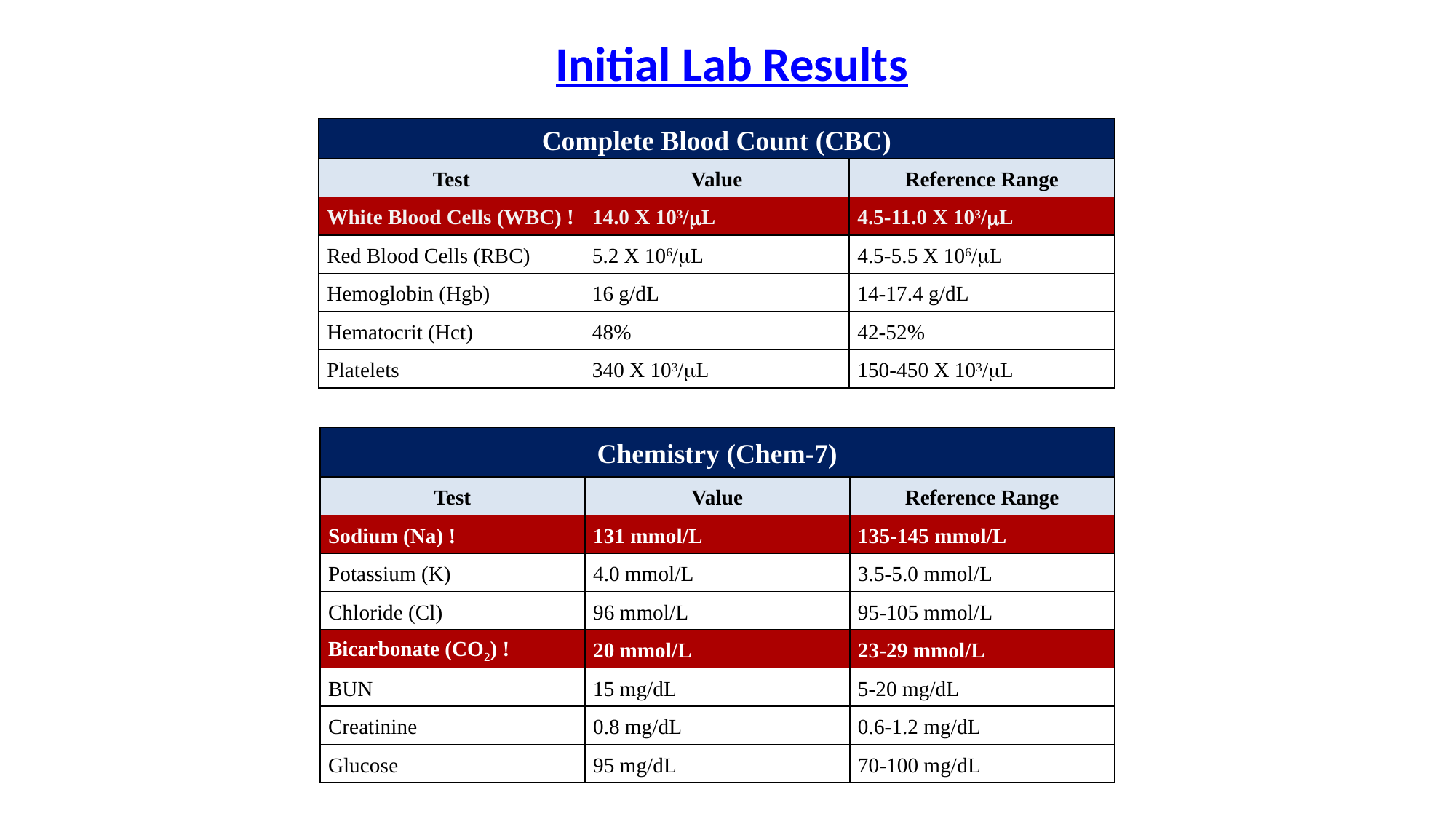

Initial Lab Results
| Complete Blood Count (CBC) | | |
| --- | --- | --- |
| Test | Value | Reference Range |
| White Blood Cells (WBC) ! | 14.0 X 103/μL | 4.5-11.0 X 103/μL |
| Red Blood Cells (RBC) | 5.2 X 106/μL | 4.5-5.5 X 106/μL |
| Hemoglobin (Hgb) | 16 g/dL | 14-17.4 g/dL |
| Hematocrit (Hct) | 48% | 42-52% |
| Platelets | 340 X 103/μL | 150-450 X 103/μL |
| Chemistry (Chem-7) | | |
| --- | --- | --- |
| Test | Value | Reference Range |
| Sodium (Na) ! | 131 mmol/L | 135-145 mmol/L |
| Potassium (K) | 4.0 mmol/L | 3.5-5.0 mmol/L |
| Chloride (Cl) | 96 mmol/L | 95-105 mmol/L |
| Bicarbonate (CO2) ! | 20 mmol/L | 23-29 mmol/L |
| BUN | 15 mg/dL | 5-20 mg/dL |
| Creatinine | 0.8 mg/dL | 0.6-1.2 mg/dL |
| Glucose | 95 mg/dL | 70-100 mg/dL |

## Slide 4
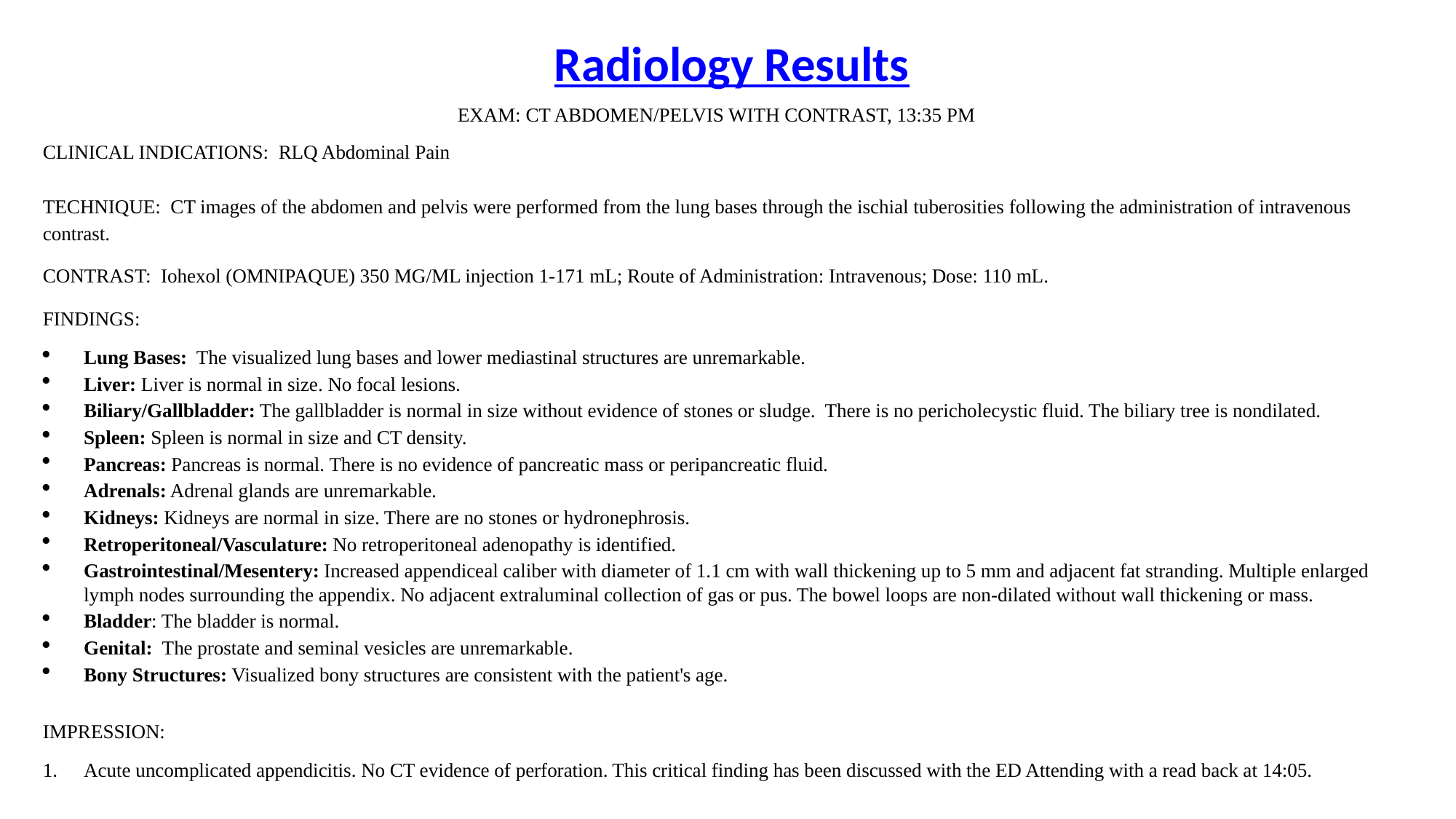

Radiology Results
EXAM: CT ABDOMEN/PELVIS WITH CONTRAST, 13:35 PM
CLINICAL INDICATIONS: RLQ Abdominal Pain
TECHNIQUE: CT images of the abdomen and pelvis were performed from the lung bases through the ischial tuberosities following the administration of intravenous contrast.
CONTRAST: Iohexol (OMNIPAQUE) 350 MG/ML injection 1-171 mL; Route of Administration: Intravenous; Dose: 110 mL.
FINDINGS:
Lung Bases: The visualized lung bases and lower mediastinal structures are unremarkable.
Liver: Liver is normal in size. No focal lesions.
Biliary/Gallbladder: The gallbladder is normal in size without evidence of stones or sludge. There is no pericholecystic fluid. The biliary tree is nondilated.
Spleen: Spleen is normal in size and CT density.
Pancreas: Pancreas is normal. There is no evidence of pancreatic mass or peripancreatic fluid.
Adrenals: Adrenal glands are unremarkable.
Kidneys: Kidneys are normal in size. There are no stones or hydronephrosis.
Retroperitoneal/Vasculature: No retroperitoneal adenopathy is identified.
Gastrointestinal/Mesentery: Increased appendiceal caliber with diameter of 1.1 cm with wall thickening up to 5 mm and adjacent fat stranding. Multiple enlarged lymph nodes surrounding the appendix. No adjacent extraluminal collection of gas or pus. The bowel loops are non-dilated without wall thickening or mass.
Bladder: The bladder is normal.
Genital: The prostate and seminal vesicles are unremarkable.
Bony Structures: Visualized bony structures are consistent with the patient's age.
IMPRESSION:
Acute uncomplicated appendicitis. No CT evidence of perforation. This critical finding has been discussed with the ED Attending with a read back at 14:05.

## Slide 5
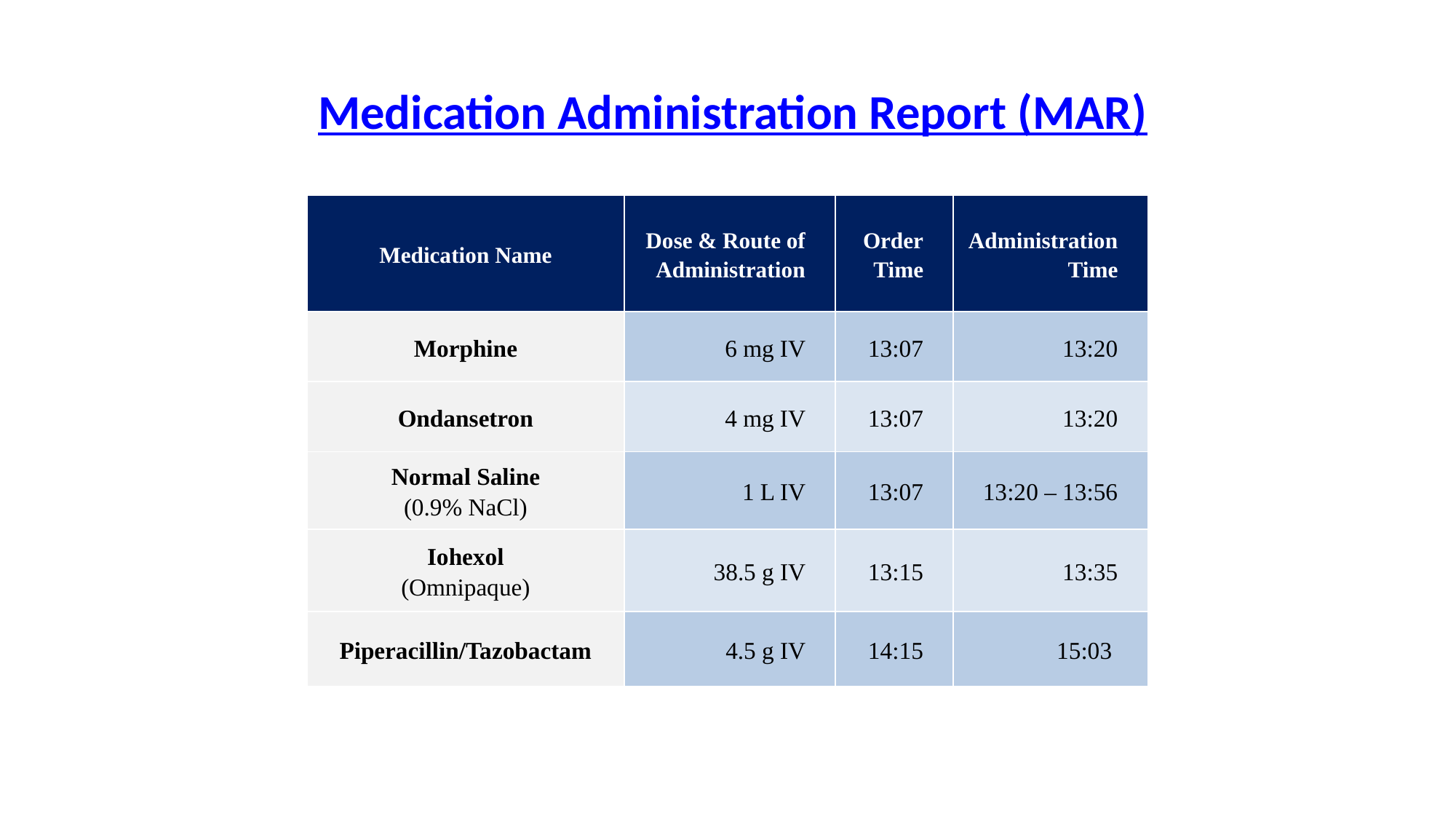

Medication Administration Report (MAR)
| Medication Name | Dose & Route of Administration | Order Time | Administration Time |
| --- | --- | --- | --- |
| Morphine | 6 mg IV | 13:07 | 13:20 |
| Ondansetron | 4 mg IV | 13:07 | 13:20 |
| Normal Saline (0.9% NaCl) | 1 L IV | 13:07 | 13:20 – 13:56 |
| Iohexol (Omnipaque) | 38.5 g IV | 13:15 | 13:35 |
| Piperacillin/Tazobactam | 4.5 g IV | 14:15 | 15:03 |

## Slide 6
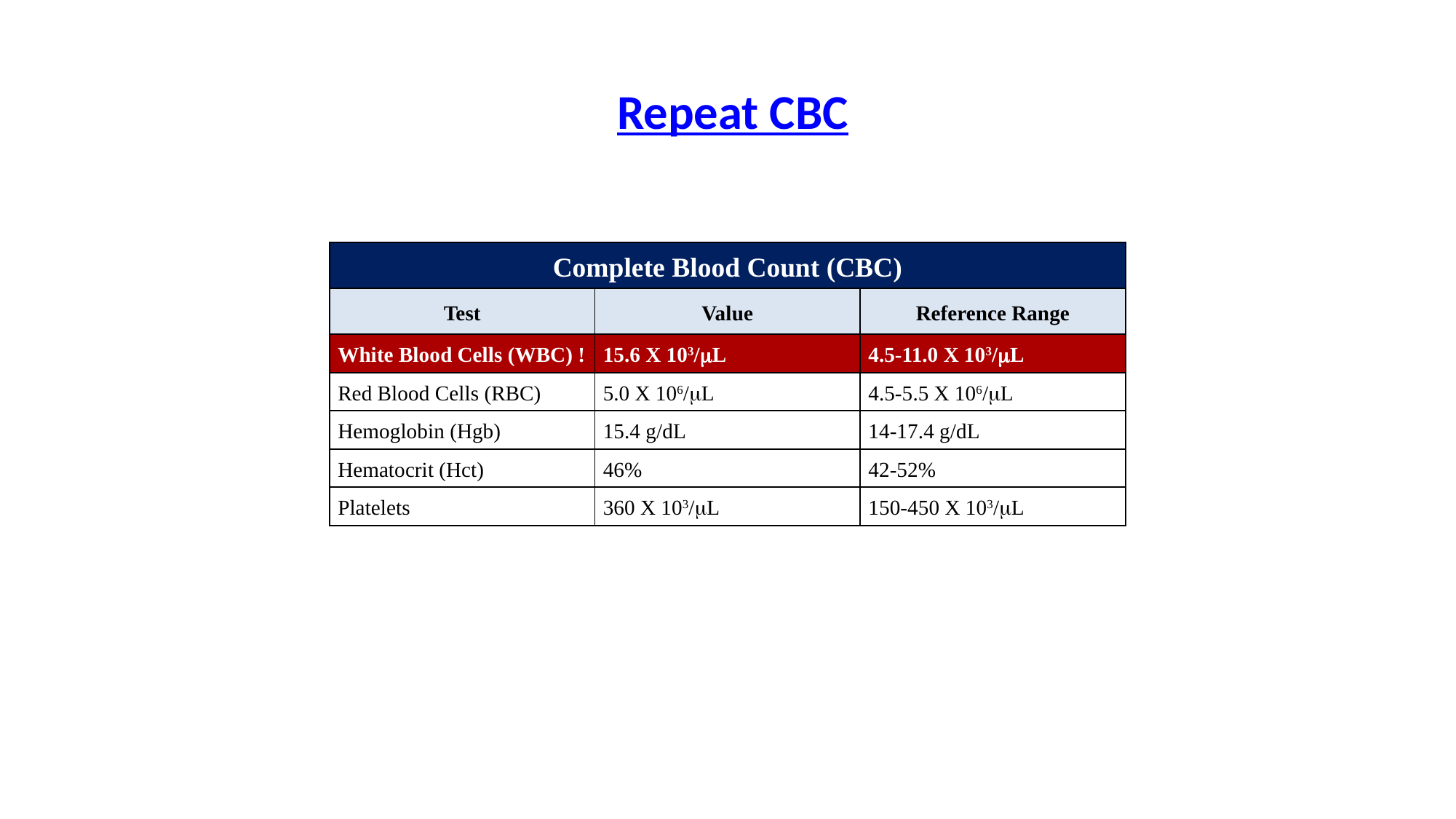

Repeat CBC
| Complete Blood Count (CBC) | | |
| --- | --- | --- |
| Test | Value | Reference Range |
| White Blood Cells (WBC) ! | 15.6 X 103/μL | 4.5-11.0 X 103/μL |
| Red Blood Cells (RBC) | 5.0 X 106/μL | 4.5-5.5 X 106/μL |
| Hemoglobin (Hgb) | 15.4 g/dL | 14-17.4 g/dL |
| Hematocrit (Hct) | 46% | 42-52% |
| Platelets | 360 X 103/μL | 150-450 X 103/μL |

## Slide 7
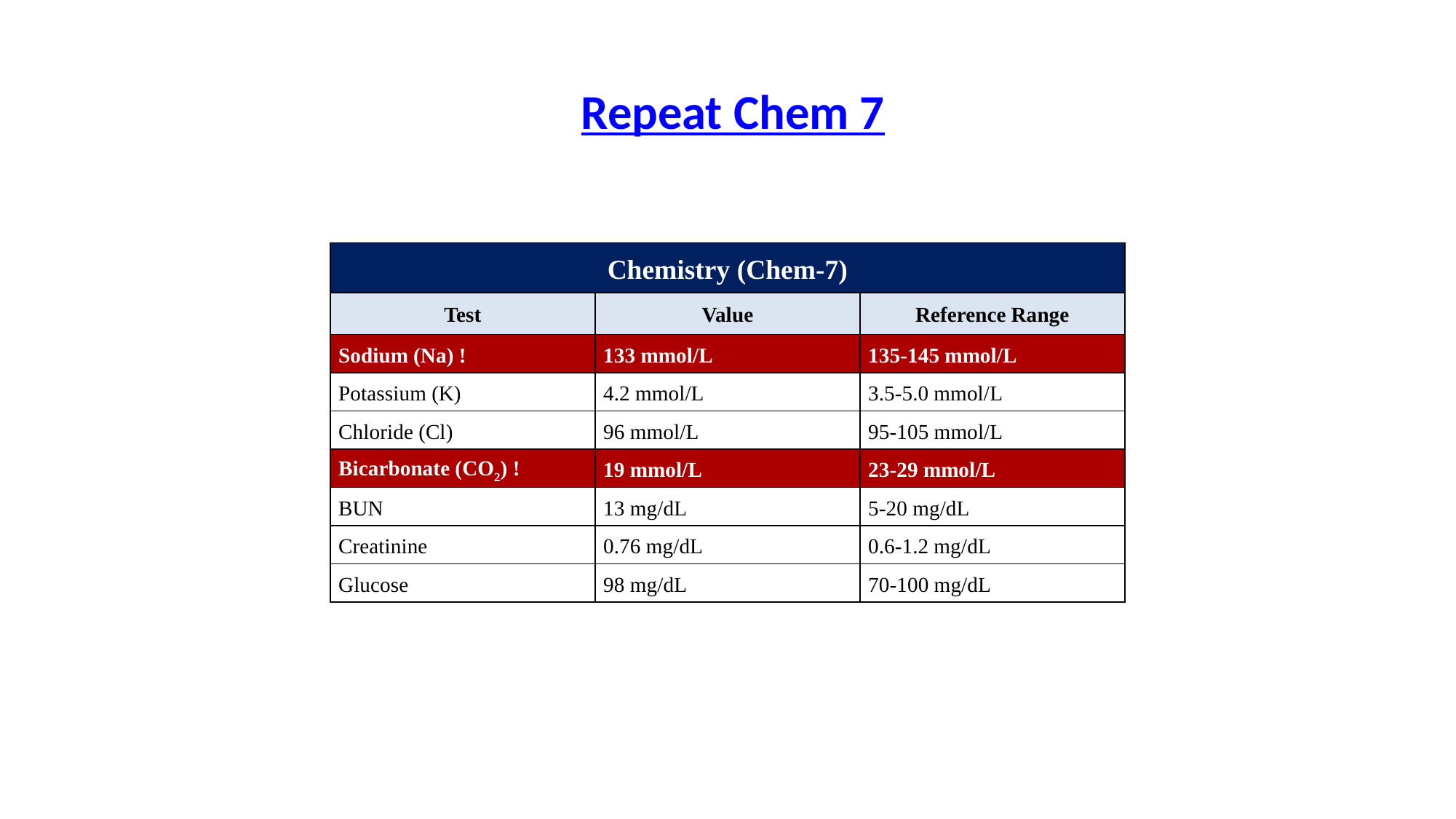

Repeat Chem 7
| Chemistry (Chem-7) | | |
| --- | --- | --- |
| Test | Value | Reference Range |
| Sodium (Na) ! | 133 mmol/L | 135-145 mmol/L |
| Potassium (K) | 4.2 mmol/L | 3.5-5.0 mmol/L |
| Chloride (Cl) | 96 mmol/L | 95-105 mmol/L |
| Bicarbonate (CO2) ! | 19 mmol/L | 23-29 mmol/L |
| BUN | 13 mg/dL | 5-20 mg/dL |
| Creatinine | 0.76 mg/dL | 0.6-1.2 mg/dL |
| Glucose | 98 mg/dL | 70-100 mg/dL |

## Slide 8
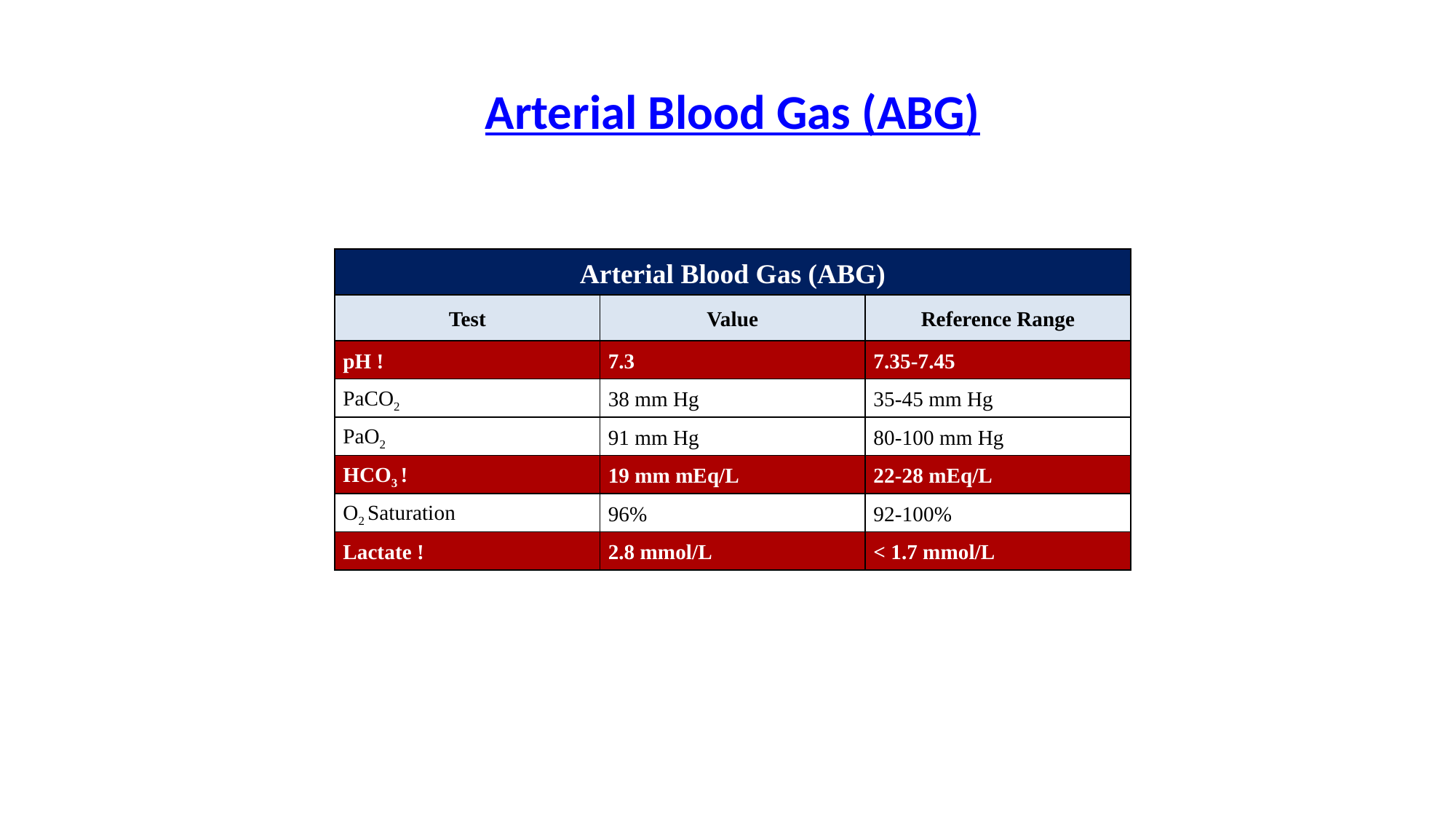

Arterial Blood Gas (ABG)
| Arterial Blood Gas (ABG) | | |
| --- | --- | --- |
| Test | Value | Reference Range |
| pH ! | 7.3 | 7.35-7.45 |
| PaCO2 | 38 mm Hg | 35-45 mm Hg |
| PaO2 | 91 mm Hg | 80-100 mm Hg |
| HCO3 ! | 19 mm mEq/L | 22-28 mEq/L |
| O2 Saturation | 96% | 92-100% |
| Lactate ! | 2.8 mmol/L | < 1.7 mmol/L |

## Slide 9
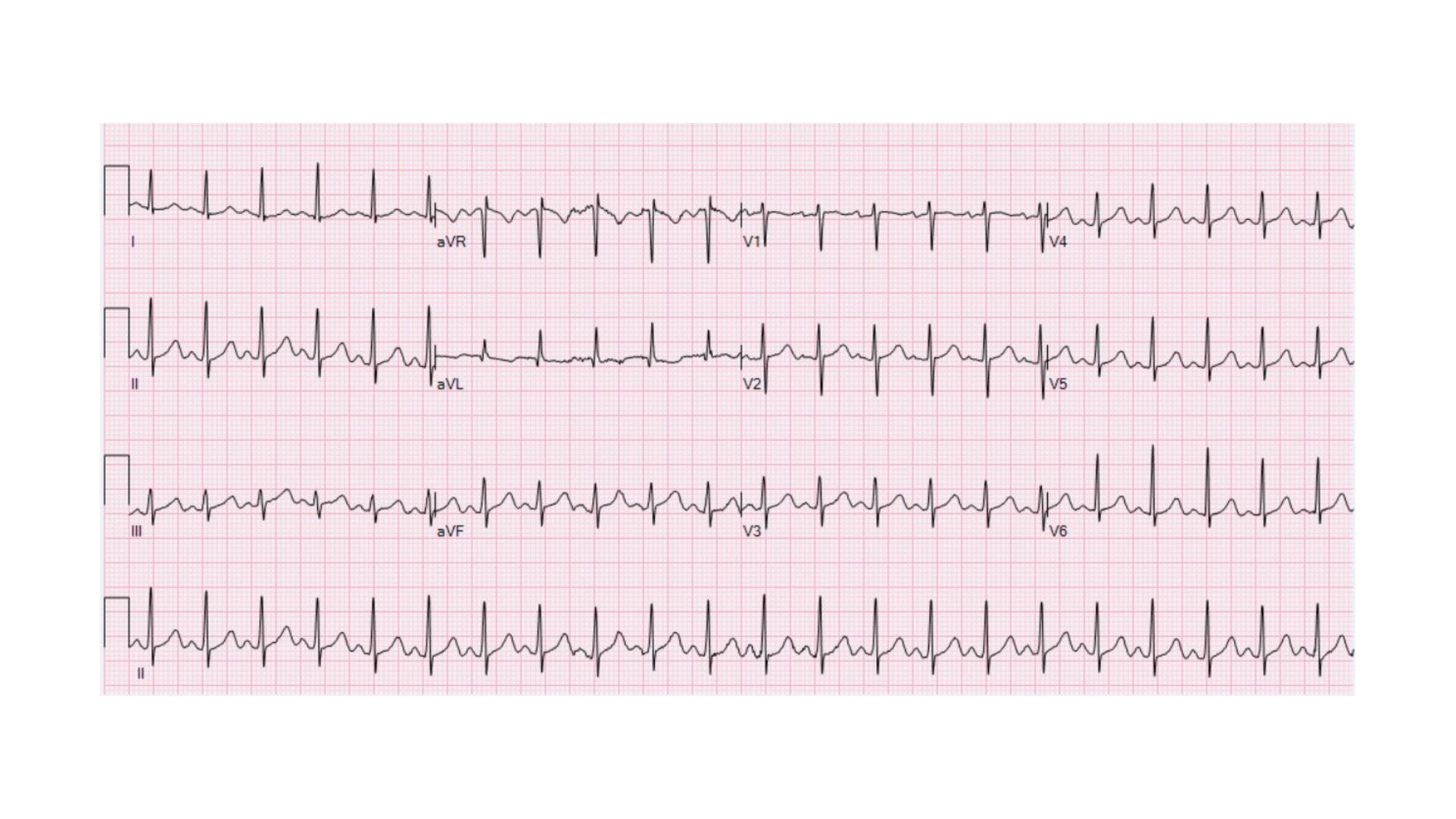

## Slide 10
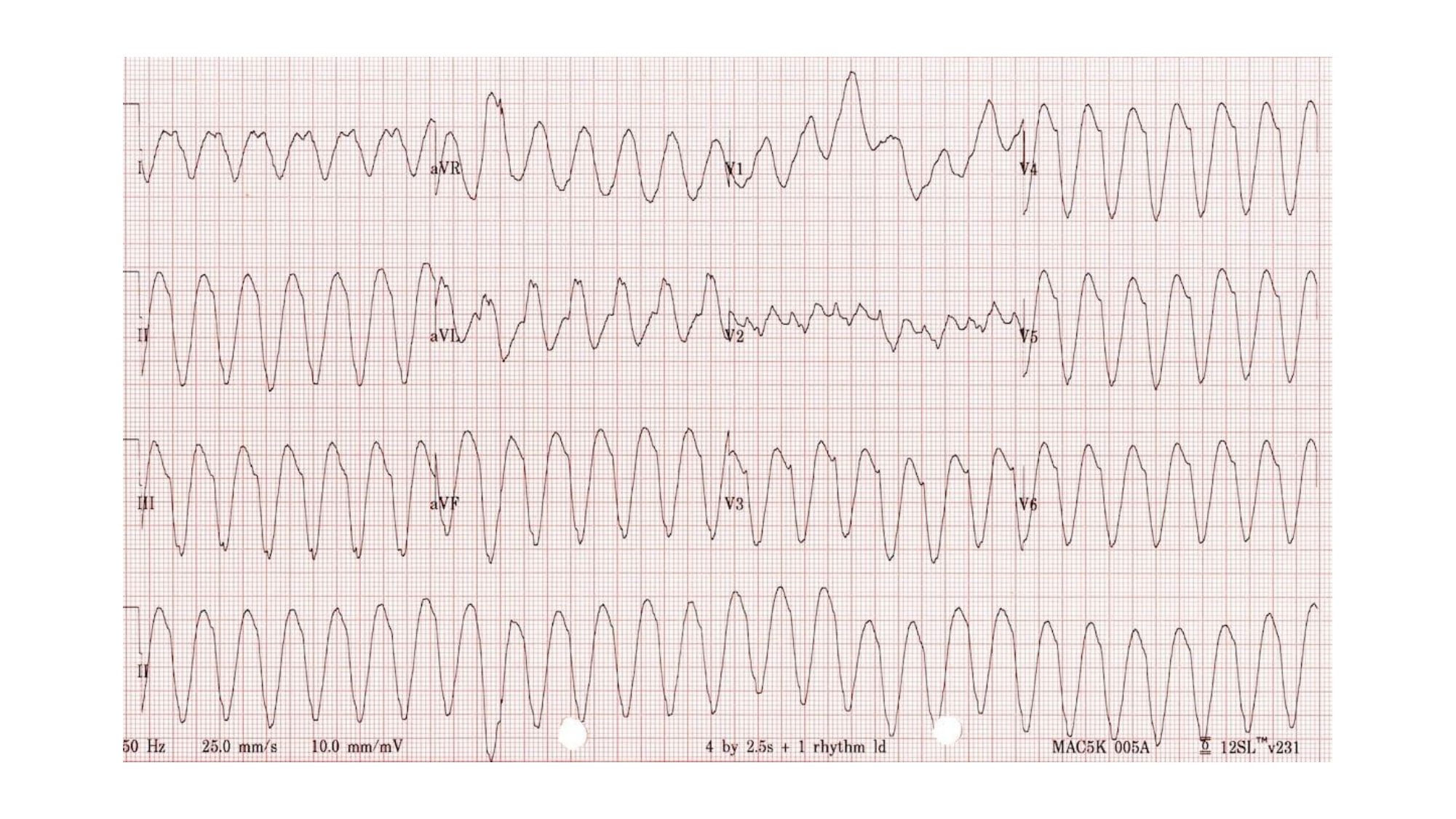

## Slide 11
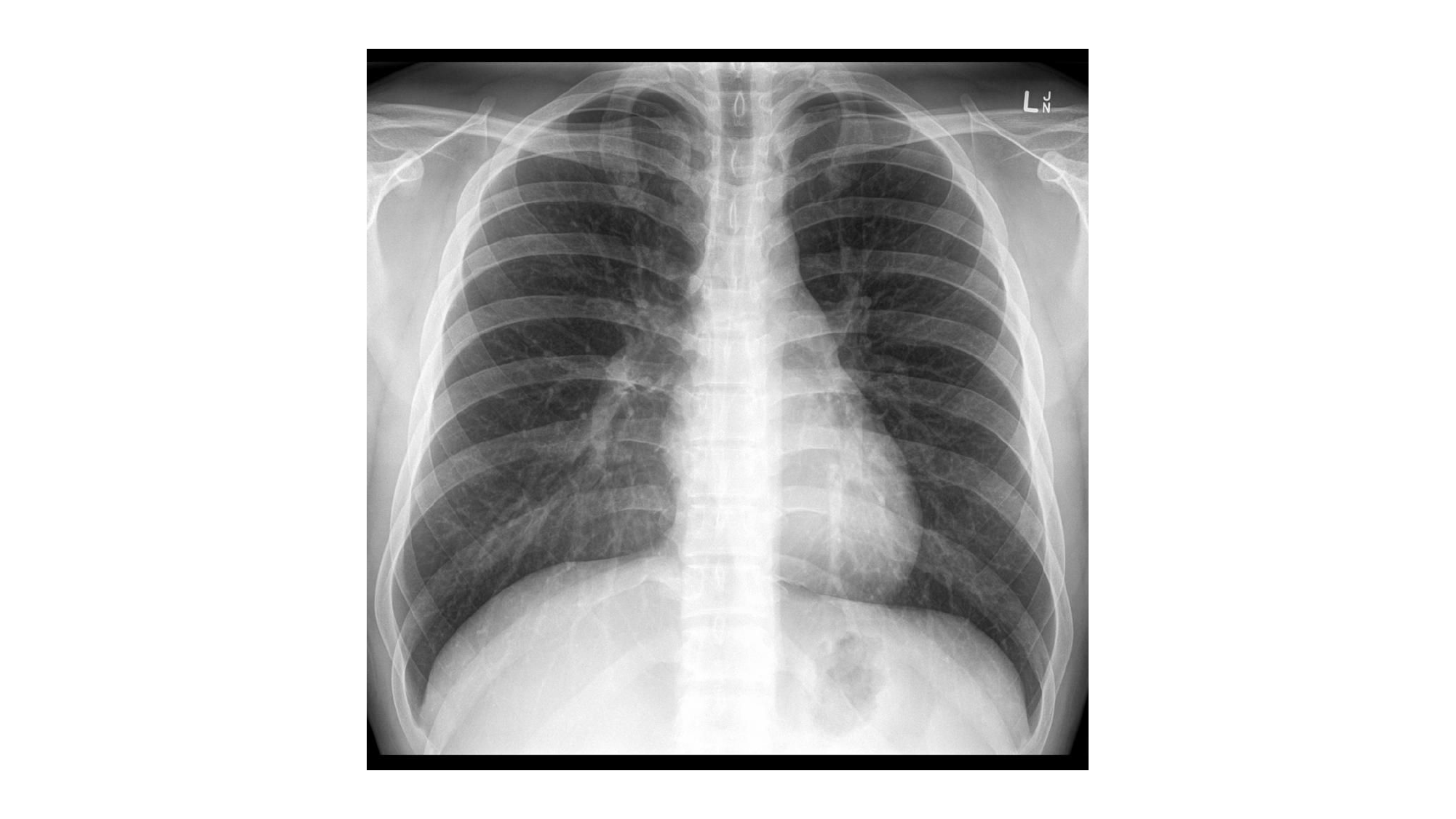

Supplement: Supplementary file 1 [file jetem-9-1-S1-supp1.ppx.pptx]
